# A tribute to our reviewers: *Arthroplasty* 2025 review and future outlook

**DOI:** 10.1186/s42836-026-00378-7

**Published:** 2026-03-16

**Authors:** Yan Wang

**Affiliations:** Department of Orthopaedics, General Hospital of Chinese People’s Liberation Army, Beijing, 100853 China

The year 2025 marked a pivotal milestone for *Arthroplasty*. We were proud to see the journal achieve an Impact Factor of 4.3, firmly securing a position in the Q1 quartile of the JCR Orthopedics category. This recognition reflects the growing trust of the global arthroplasty community in our platform and marks a significant step forward in our mission to become a leading voice in the field.

However, bibliometric metrics only tell half the story. The true engine behind this success is our peer review process. While high-quality authorship provides the fuel, it is the rigorous, constructive, and often tireless work of our reviewers that refines the research and drives our scientific standards.

In 2025, *Arthroplasty* received a record-breaking number of submissions. To ensure the rigorous assessment of this growing volume of research, 271 reviewers from across the globe contributed over 570 reports. Your critical insights ensured that only the most robust science made it to publication. To strictly adhere to data privacy regulations, we have elected not to publish a comprehensive list of reviewer names this year. However, we want to express our deepest gratitude to each of you. You are the silent guardians of our scientific integrity.

## Looking ahead: efficiency, innovation, and community

As we move into 2026, *Arthroplasty* is not resting on its laurels. We are launching several initiatives to better serve our community, starting with a significant upgrade to our Reviewer Board (RB). Recognizing the vital role of our core reviewers, we invite dedicated experts to join this expanded team to help guide the journal’s future. RB members will enjoy exclusive privileges, including official service certification for career development, expedited editorial assessment for their own submissions, and priority consideration for Youth Editorial Board nominations. Additionally, top-performing reviewers will be eligible for annual APC discount awards.

Furthermore, we remain committed to optimizing our workflow, aiming for a faster peer-review timeline without compromising quality. Scientifically, we will place a renewed focus on innovation, specifically encouraging submissions in emerging fields such as Robotics, AI applications in surgery, and Personalized Arthroplasty, bridging the gap between cutting-edge technology and clinical reality.

If you contributed a review in 2025 and would like to receive an official Peer-Review Certificate for your records, or if you are interested in joining our expanded Reviewer Board, please contact the Editorial Office at [edit-group@asiaarthroplasty.com]. It would be our honor to recognize your contribution.

## Data Availability

Not applicable.

